# Tuberculosis Epidemiology and Selection in an Autochthonous Siberian Population from the 16^th^-19^th^ Century

**DOI:** 10.1371/journal.pone.0089877

**Published:** 2014-02-26

**Authors:** Henri Dabernat, Catherine Thèves, Caroline Bouakaze, Dariya Nikolaeva, Christine Keyser, Igor Mokrousov, Annie Géraut, Sylvie Duchesne, Patrice Gérard, Anatoly N. Alexeev, Eric Crubézy, Bertrand Ludes

**Affiliations:** 1 Molecular Anthropology and Image Synthesis (AMIS) Laboratory, UMR 5288, CNRS, University of Toulouse (Paul Sabatier 3); University of Strasbourg, Toulouse, France; 2 Cultural History Centre of Contemporary Societies (CHCSC), University of Versailles Saint-Quentin-en-Yvelines, Versailles, France; 3 Laboratory of Molecular Microbiology, St. Petersburg Pasteur Institute, St. Petersburg, Russia; 4 Institute of Legal Medicine, University Paris Descartes, Paris, France; 5 Institute of the Humanities and the Indigenous Peoples of the North, Siberian Branch of the Russian Academy of Sciences, Yakutsk, Russia; University of Florence, Italy

## Abstract

Tuberculosis is one of most ancient diseases affecting human populations. Although numerous studies have tried to detect pathogenic DNA in ancient skeletons, the successful identification of ancient tuberculosis strains remains rare. Here, we describe a study of 140 ancient subjects inhumed in Yakutia (Eastern Siberia) during a tuberculosis outbreak, dating from the 16^th^–19^th^ century. For a long time, Yakut populations had remained isolated from European populations, and it was not until the beginning of the 17^th^ century that first contacts were made with European settlers. Subsequently, tuberculosis spread throughout Yakutia, and the evolution of tuberculosis frequencies can be tracked until the 19^th^ century. This study took a multidisciplinary approach, examining historical and paleo-epidemiological data to understand the impact of tuberculosis on ancient Yakut population. In addition, molecular identification of the ancient tuberculosis strain was realized to elucidate the natural history and host-pathogen co-evolution of human tuberculosis that was present in this population. This was achieved by the molecular detection of the IS*6110* sequence and SNP genotyping by the SNaPshot technique. Results demonstrated that the strain belongs to cluster PGG2-SCG-5, evocating a European origin. Our study suggests that the Yakut population may have been shaped by selection pressures, exerted by several illnesses, including tuberculosis, over several centuries. This confirms the validity and necessity of using a multidisciplinary approach to understand the natural history of M*ycobacterium tuberculosis* infection and disease.

## Introduction

Tuberculosis (TB) is one of the oldest human diseases in the world, and even today, according to World Health Organization (WHO), it is second only to HIV/AIDS as the greatest killer worldwide due to a single infectious agent (http://www.who.int/mediacentre/factsheets/fs104/en/). It is caused by a group of phylogenetically closely related bacteria, collectively known as the *Mycobacterium tuberculosis* Complex (MTBC). Understanding host-pathogen co-evolution in TB will help to develop better tools and strategies to control its expansion [1]. Concerning the host, recent studies have shown that the long-term association between MTBC and its human host has shaped the biology and epidemiology of human TB. Although several heritability, linkage and candidate gene association studies have investigated TB susceptibility, the exact causative genetic variants have not been characterized [1], [2]. Different factors and biases could explain why some associations could not be validated [1], [2], but it is important to note that all human TB genetic studies are impeded by our inability to determine the degree of exposure to *M. tuberculosis* [3], and the different degrees of natural selection on past populations. Concerning the pathogen, most of the strains that have infected past populations are unknown [4].

In this context, identifying ancient strains of TB and evaluating the strength and role of natural selection, in terms of mortality, will help to elucidate the natural history and host-pathogen co-evolution of human tuberculosis. We propose such an approach for a sample of 140 frozen bodies, dating from the 16^th^–19^th^ century, from Yakutia in Eastern Siberia. For a long time this region had been isolated from contact with European populations, and it was not until the beginning of the 17^th^ century that first contacts were made with Slavic Russians. The value of this sample, apart from it representing a naïve population, is also related to its exceptional state of preservation under permafrost conditions, which has allowed the detection, not only of human sequences [5], but also of the bacterial and viral genetic material present in each subject [6], [7]. The study takes a multidisciplinary approach [2], [8]; we examine historical data, conduct macroscopic observations for diagnosis of tuberculosis, and conduct genetic analyses in order to test the possibility to amplify *Mycobacterium tuberculosis*, and thus, confirm the presence of tuberculosis, and to identify the ancient strains involved. Finally, we study the chronological distribution of tuberculosis cases to determine how the disease spread.

## Materials and Methods

### Archaeological samples

**Figure 1:**
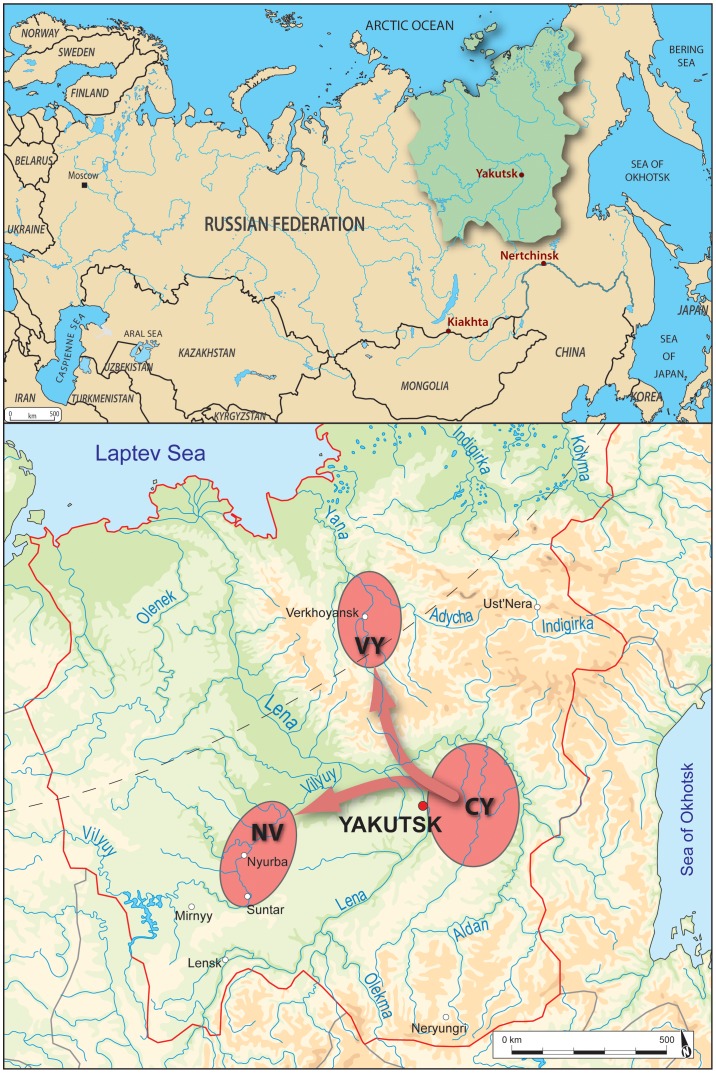
Map of archaeological regions of excavations. VY: Verkhoyansk Yana area; CY: the Central Yakutia area and NV: Nyurba Vilyuy area.

**Figure 2:**
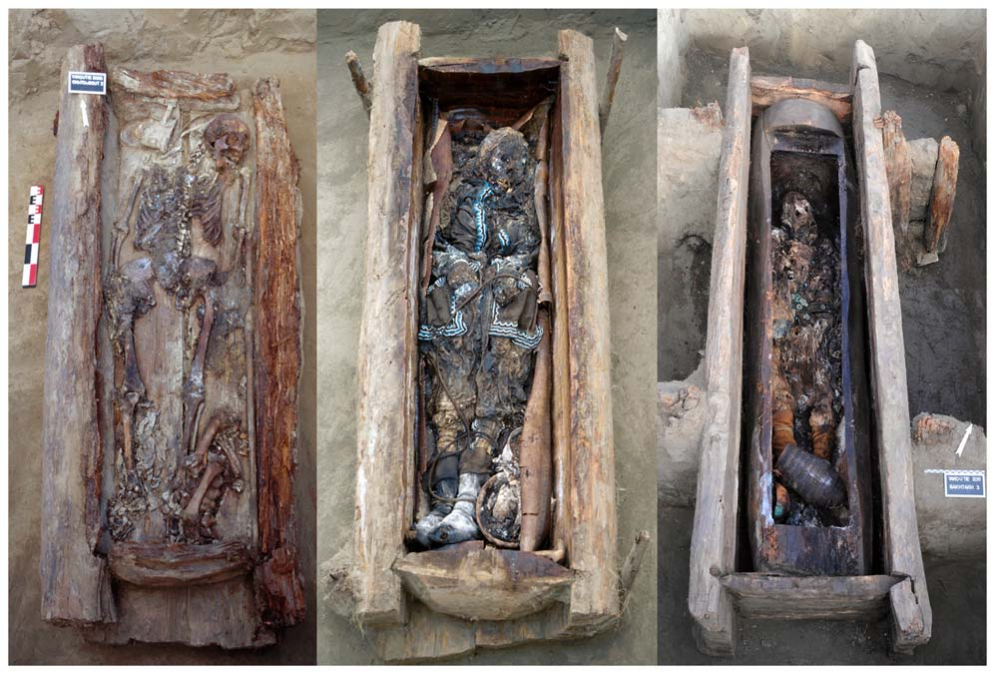
Graves of three frozen bodies from Yakutia. Bodies were autopsied and diagnosed as having bone tuberculosis: Graves of Okhtoubout 2, Kyys Ounhouogha and Bakhtakh 3 respectively.

From 2002 to 2012, a French-Russian team studying the evolution of Yakut populations conducted excavations in the regions of central Yakutia, Vilyuy and Verkhoyansk; ecologically different areas that Yakuts inhabited when the Russians first arrived in 1630. We have shown that contact between regions were frequent, related to trade and the movement of people (Figure 1; [9]). Excavations identified 134 isolated archaeological graves, separated by distances of one to 900 km (Figure 1; [4]). One hundred and forty subjects were unearthed (132 graves contained one subject, 2 graves contained multiple burials); all bodies were frozen due to inhumation in the permafrost (Figure 2) and so this sample represents the largest frozen mummy sample in the world. The wooden graves were dated precisely to the 16^th^ to the early/mid 19^th^ century by dendrochronological analysis, the burial construction technique and the artifacts held inside. During the period studied [9], the sex ratio of the sample was balanced, but child burials remained rare until the spread of Christianity in the early 19^th^ century [4]. Information on the age and sex of subjects, along with their datations and geographic locations are presented in Table 1.

**Table 1:** Repartition of sampled subjects by geographical regions, periods of time, age and sex.

			Age of subject						Datation			
Geographical Regions	Study Year(s)	N	New Born	Child	Adolescent	Adult male	Adult female	Sex Unknown	16–17^th^C	18^th^C	19^th^C	Datation Unknown
Central Yakutia (CY)	2003–2006 2009	96	4	21	4	37	28	2	14	58	24	0
Nyurba Vilyuy (NV)	2007–2008	23	1	5	1	9	7	0	2	10	10	1
Verkhoyansk Yana (VY)	2010–2011	21	2	2	5	7	5	0	2	16	3	0
Total		140	7	28	10	53	40	2	18	84	37	1

N: number of subjects; New Born: 1–12 months; Child: 13 months-9 years; Adolescent: 10–19 years; Adult: > 19 years; C: Century.

### Historical data

Historical data available on tuberculosis in Russia [10]–[13] was reviewed critically. Most data dated from the 19^th^ and 20^th^ centuries; documents were either old or were written during the Stalin era, and so had been interpreted or censured [14].

### Pathology

Each subject was autopsied according to the forensic medicine guidelines for bodies presenting preservation of soft tissue by freezing and/or natural mummification. All the bone and tissue lesions (when remaining) were photographed, recorded and sampled. Lesions were studied in different organs, soft tissues and pieces of skeleton by the standard methods of forensic medicine. Paleopathology was studied for certain subjects, by radiological and histological examinations [5], [6], [15]–[17]. A full radiological examination was made on one subject [4], [18].

### Diagnosis of bone tuberculosis using macroscopic data

The diagnosis of bone tuberculosis was based on observations of bone characteristics considered to be tuberculosis lesions in children and adults during pre-antibiotic times, as well as in more recent times [19]–[24]. Tuberculosis shows in its clinical evolution specific and frequent bone localizations [23], [25], [26] whose characteristics include: different lytic lesions localized on the vertebrae (spondylitis leading to Pott's disease), long bones and joints, and cold abscesses on other skeletal bones. For each frozen body radiological and forensic findings, including bloody pleurisy and infectious lesions of the spine, sacroiliac joint, or pelvis, were necessary to conclude that death was due to a disseminated infection.

### Ancient DNA analysis

#### Precautions against contamination

Samples were handled taking into consideration the critical issue of pre-laboratory contamination in ancient DNA (aDNA) studies. Ancient vertebrae were sampled by a forensic scientist within a few minutes of being exposed, and were transported to the laboratory in individual bags and stored in controlled conditions until the molecular investigations. In the laboratory, the precautions recommended when working with ancient DNA were followed: sample preparation and all analytical steps prior to the first PCR amplification were conducted in a specialized aDNA laboratory where no work with modern DNA (human or *M. tuberculosis*) is permitted [7]. This pre-PCR area is physically separated from the post-PCR laboratory. Within the aDNA laboratory, strict precautions regarding cleaning, equipment, reagents and clothing were taken [27]. Concerning analysis, blank extraction and PCR controls were always included; at least two independent extractions were performed from each sample and each PCR reaction was reproduced twice to monitor cross-contamination.

#### Sample preparation and DNA extraction

To minimize contamination, the outer surface of each vertebra was abraded using a sanding machine (Dremel). The vertebral body of each vertebra was then reduced to fine bone powder in liquid nitrogen using a 6870 SamplePrep Freezer Mill^®^ (Fischer Bioblock). DNA extraction was performed on 250 mg of bone powder using two different silica filter column-based procedures as described previously [27], [28].

#### IS6110 MTBC analysis

Firstly, all samples were tested for the presence of MTBC DNA by targeting a specific region of the repetitive element IS*6110*, an insertion element used for the identification of the MTBC strains. The commonly used two-tube nested PCR, producing an 123 bp outer product and a 92 bp inner product was performed using primer pairs published previously ([29], [30]; Table S1). PCRs were optimized for the analysis of aDNA. The first PCR was conducted in a final volume of 50 µL containing 1X of Buffer Gold (Applied Biosystems), 1.5 mM of MgCl2, 0.2 mM of each dNTP, 0.5 µM of each external primer, 0.2 mg/ml of BSA (Sigma-Aldrich), 1.25 U of AmpliTaq Gold Polymerase (Applied Biosystems) and 5 µl of DNA extract. Thermal cycling conditions were 95°C for a 5 min denaturation step, followed by 40 PCR cycles at 94°C for 1 minute, 66°C for 1 minute, 72°C for 1 minute and final extension at 72°C for 7 minutes. The second PCR was conducted on 1 µL of PCR products using the nested primers in the same conditions as described above, but with only 25 cycles with an annealing step at 58°C. The specificity of the PCR products of expected size was confirmed by direct sequencing in both directions using the Big Dye Terminator 3.1 Kit (Applied Biosystems), according to manufacturer's protocol. Sequencing products were separated on an ABI Prism 3100 or 3500 Genetic Analyzer (Applied Biosystems) and the resulting sequences were edited using the software Sequencher v.4.7 (Genecodes).

#### SNP MTBC genotyping

SNP genotyping further characterized the samples that successfully amplified the IS*6110* region. These samples were first examined for the presence of SNPs at four positions: position 1410 of *gyrB* gene (C→T in *M. bovis* and *M. bovis* BCG), three positions in codon 95 of *gyrA* gene, and codons 203 and 463 of *katG* gene whose combination provides discrimination between the three principal genetic groups (PGG1 to PGG3), as defined by Sreevatsan et al. [31]. Based on the results of this first genotyping assay, four additional SNPs were tested to determine specific downstream phylogenetic groupings to gain further resolution. SNP genotyping was performed using the SNaPshot minisequencing approach (Applied Biosystems) and primers described previously [32]. As seen in Table S1, the primers designed validated the first step of multiplex PCR1 (mPCR1), followed by SBE1 to validate species and principal genetics groups, as discussed above. A second step of multiplex PCR2 (mPCR2) followed by SBE2 was realized to validate lineage specific SNPs. The SNapShot technique was performed from the first classical PCR amplification; the sizes of the amplicons ranged from 72 to 150 bp with mPCR1, and 81 to 141 bp with mPCR2. In the second step, the minisequencing primers were tailed at the 5′end with a non-homologuous sequence [32] and poly(C), if necessary, to produce extension products that ranged in length from 28 to 76 nucleotides (nt) with SBE1, and from 31 to 73 nt with SBE2 (see Table S1). Thus, extension products differed in length from each other by at least 6 nt to allow sufficient separation by capillary electrophoresis. All conditions of mPCR, SBE and capillary electrophoresis are given in [32]. The data obtained from purified SBE products were analyzed and the alleles were automatically called by the Gene Mapper ID software (v.3.2.1 Applied Biosystems). SNaPshot results were validated by directly sequencing segments containing SNP positions for some selected amplicons obtained from mPCR1 when amplifications were positive. For the SNapShot technique, however, targeted segments are more or less small, and so the sequenced segments are small in certain cases.

#### Human DNA amplification:

Human autosomal STR typing was performed using the AmpFlSTR Identifiler kit (Applied Biosystems), following the manufacturer's recommendations, to test for the presence of amplifiable DNA in extracts and to assure that the PCR reaction was not inhibited.

### Statistical analysis

The palaeoepidemiological study was undertaken after grouping the TB and non-TB subjects into time periods [33]. Prevalence was calculated as the number of individuals presenting the disease divided by the number of individuals in the study population. Crude Prevalence Rate (CPR) was calculated for each time period sub-unit: the number of individuals with the disease was divided by the number of individuals in each period sub-unit. The Chi-squared (χ^ 2^) test was calculated using Statistica v.6.0 Graphs were realized with Illustrator CS4.

## Results

### The archaeological samples

**Figure 3:**
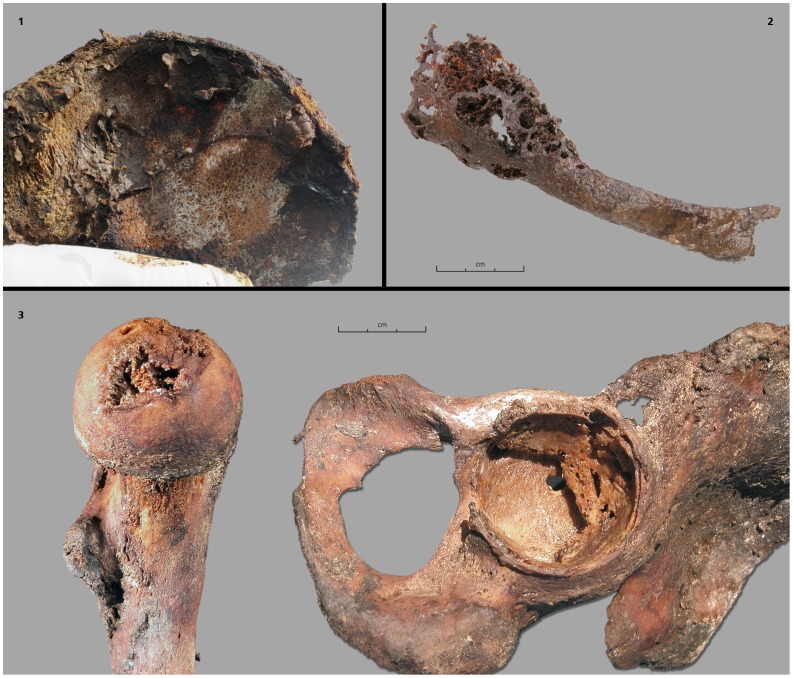
Bone tuberculosis lesions. 1: Bakhtakh 3: the innominate bone (right iliac wing) with periostal reaction; 2: Kous Tcharbit: infectious lesion costal; 3: Bouogaryma 2: tuberculous involvement of the left hip.

The sample contained significantly less children than adults (Table 1; χ^2^ = 8.35, p<0.005), and for adults there was no significant difference between the frequency of sexes (χ^2^  = 0.91, p = 0.18). Cases of tuberculosis were found in each of the three regions (Table 2). It seems that for some males Y-chromosomal STR and autosomal STR lineages may have been buried preferentially over other lineages, as a function of their higher social status [9]. Female autosomal STR and mtDNA lineages showed great variability [5]. Therefore, this sample gives a representative picture of the state of the population in studied periods. There are two limitations of the sample: 1/The small sample size leads to large confidence intervals (Table S2) when exploring variation in the subjects with tuberculosis; and 2/Modeling the zero point upstream of the epidemic (Figure 3) is difficult to determine accurately, although historical data does indicate the beginning of 17^th^ century [10], [14]. Note, however, that for a population of the past, the quantity and quality of subjects studied is exceptional.

**Table 2:** Crude Prevalence Rate (CPR) of cases of tuberculosis according to time period and geographic area.

	16–17^th^C (n = 18)	18^th^C 1st half (n = 50)	18^th^C 2nd half (n = 34)	19^th^C (n = 37)
Global (13)	11.1% (2/18)	14% (7/50)	11.7% (4/34)	(0/37)
Central Yakutia (7)	14.3% (2/14)	11.1% (4/36)	4.5% (1/22)	(0/24)
Verkhoyansk Yana (3)	0/2	25% (2/8)	12.5% (1/8)	(0/3)
Nyurma Vilyuy (3)	0/2	16.6% (1/6)	25% (2/4)	(0/10)

C: century

### Diagnosis and frequency of tuberculosis from macroscopic data

During the excavation, the bodies and bones were observed to be well-preserved, even for newborns. Subjects dated from the 16^th^ to 19^th^ century. 13/140 subjects, presented skeletal injuries compatible with the bony involvement of tuberculosis. These subjects were over the age of one and originated from three geographic areas: 1/Central Yakutia area (seven subjects); 2/Verkhoyansk (three subjects); and 3/Nyurba Vilyuy (three subjects) (Tables 2 and 3). All lesions are in agreement with the morphological diversity of tuberculosis lesions during the pre-antibiotic era [23].

**Table 3:** Details of all subjects identified with bone tuberculosis lesions and tested by genetic analysis.

Burial site	Excavation	Location (Ulus)	Datation	Age	Sex
Batta Tcharana	2005	Tattinsky, Central yakutia	17^th^ (end)	30–60 years	M
Kous Tcharbyt	2005	Tattinsky, Central Yakutia	18^th^ (first half)	40–60 years	M
Bouogaryma 1	2005	Tattinsky, Central Yakutia	17^th^ (end)	40–60 years	M
Bouogaryma 2	2005	Tattinsky, Central Yakutia	18^th^ (second half)	mature	F
Okhtoubout 2	2005	Churapchinsky, Central Yakutia	18^th^ (first half)	mature	F
Kyys Ounhouogha	2006	Churapchinsky, Central Yakutia	18^th^ (1728 dendrochronology)	18–23 years	F
Odjuluun 2	2006	Churapchinsky, Central Yakutia	18^th^ (1741 dendrochronology)	30–35 years	F
Atakh	2007	Nyurbinsky, West Yakutia	18^th^ (first half)	U*	M
Istiing Tumula	2007	Nyurbinsky, West Yakutia	18^th^ (second half)	mature	F
Ougout Kuel 1	2008	Suntarsky, West Yakutia	18^th^ (second half)	15–17 years	U*
Buguyekh 3	2010	Verkhoyansky, North Yakutia	18^th^ (first half)	15–18 years	M
Uettekh	2010	Verkhoyansky, North Yakutia	18^th^ (second half)	11–17 months	U*
Bakhtakh 3	2011	Verkhoyansky, North Yakutia	18^th^ (first half)	mature	M

U*: Unknown

Subjects dated from different time periods: two cases were prior to the 18^th^ century but after 1689, seven cases from the early 18^th^ century and four cases from the late 18^th^ century. None of the 37 subjects found in Christian-type burials from the 19^th^ century presented bone tuberculosis lesions (Figure 2). An older male subject, Bakhtakh 3, presented multiple bone tuberculosis lesions, including a pathological fracture of the femoral neck and numerous lesions of innominate bone, suggesting a condition of advanced tuberculosis (Figure 3.1).

**Figure 4:**
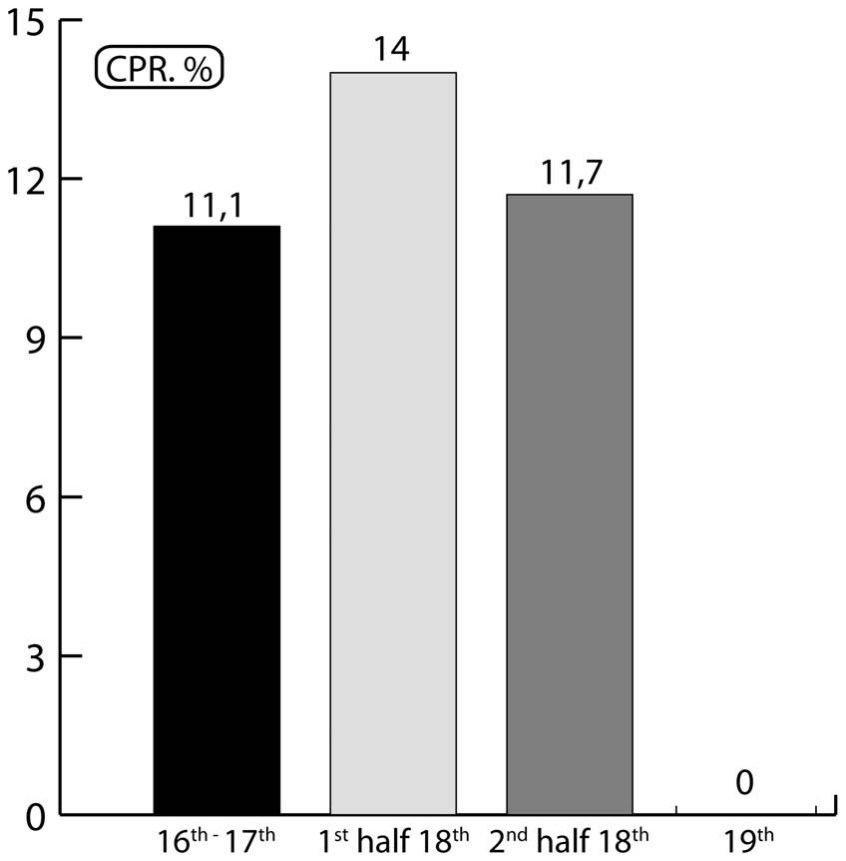
Temporal distribution of bone TB cases in Yakutia: evolution of CPR (%). Data values are presented in Table S1 (IC 95%).

CPR of bone lesions was 9.3% (13/140) for the studied population as a whole (from 16^th^ to 19^th^ centuries). For the most ancient cases to those from the end of the 18^th^ century, CPR varied from 11.1% (2/18) to 14% (7/50) (early 18^th^ century), and 11.7% (4/34) (late 18^th^ century). For subjects prior and during the 18^th^ century, CPR was 12.7% (13/102). For subjects from the 18^th^ century only, CPR was 13.1% (11/84; see Figure 4).

### DNA analysis

#### Information on blank controls, reproducibility and consensus criteria

All samples were extracted with blank controls. On average, two to four independent extractions were performed for each of the 13 samples (Table 3). These blank controls followed all the steps for MTBC DNA amplification, PCR, sequencing and SNapShot techniques. All blank controls were negative for all steps and for all samples. For sequencing, all samples with positive amplifications were sequenced for both strands and for each extract. For IS*6110*, both the forward and reverse segments obtained were sequenced for each positive sample. For SNPs detected by SNapShot, target segments were sequenced for both strands for two to four different extracts from each sample when mPCR1 results were positive. Reproducibility of sequencing was obtained for at least two or more strands (forward and/or reverse) for each positive sample, including different extracts. Reproducibility of the SNapShot genotyping technique was performed between two and four times. For sequencing, consensus sequences were realized from at least two sequences obtained for each sample. Thus, target SNPs were assigned by at least two electrophoregrams, with unequivocal peaks (see Table S3 for final sequences). The blank controls also followed all the steps for the amplification human DNA [5].

#### Human DNA amplification

Human autosomal STR typing was successfully performed to demonstrate that no PCR inhibition had occurred in our DNA samples [5]. Negative amplifications of MTBC DNA were not caused by inhibitions of PCR reactions, but were most probably due to differential DNA conservation.

#### MTBC DNA amplification

All 13 TB subjects buried in the 17^th^ and 18^th^ centuries were analyzed for MTBC DNA. Sampling was made from thoracic and lumbar vertebrae, which presented lytic lesions (Table 3). The presence of MTBC DNA was confirmed by the detection of the IS*6110* sequence; DNA fragments of expected size were observed repeatedly in the vertebrae samples recovered from these four burials: three adults (Batta Tcharana, Odjuluun 2, Atakh) and one adolescent (Buguyekh 3, 15–18 years old; Table 4). Two subjects were from central Yakutia from the late 17^th^– early 18^th^ century (1741 by dendrochronology). The other two subjects were from Nyurma Vilyuy and Verkhoyansk Yana, buried in the late 18^th^ century. As revealed by direct sequencing, these fragment sequences were identical to that of the laboratory strain *M. tuberculosis* H37Rv (NC_000962.3; data not shown). These four samples were always positive by single-stage PCR and nested PCR. The remaining samples repeatedly failed to yield PCR products of the expected size, even after multiple extraction and amplification attempts. A failure to amplify this region was also observed for blank controls. For three subjects, SNP typing by SNaPshot (as described in Bouakaze et al. [32]) was carried out to compare the three TB strains, which are geographically and temporally distant. As shown in Table 4, SNP analysis failed for one sample (Buguyekh 3). None of the samples that were analyzed successfully for the *gyrB*(1410) position gene had the T allele characteristic of the *M. bovis* species, indicating that the three individuals were not infected by a *M. bovis* strain (Table 4). Analysis of positions *katG* ^203^, *katG* ^463^ and *gyrA* ^95^ revealed that these samples were infected by *M. tuberculosis* strains belonging to PGG2 (Table 4). To further characterize the strain lineages that infected these individuals, we analyzed four additional SNP positions, when sufficient DNA extract was available. These included positions 1977, 3352929, 2460626 and 232574 in the H37Rv genome, as they enabled the PGG-2 strain to be further divided into four genetic groups: SCG-3c, SCG3-b, SCG-4 and SCG-5. The nucleotides at the targeted positions could be unambiguously and reproducibly determined for only one sample (Odjulunn 2), revealing the presence of a *M. tuberculosis* strain belonging to PGG-2/SCG-5 (Table 4).

**Table 4:** Summary of SNP genotyping data obtained from IS*6110*-positive samples.

				SNP genotyping							
Burial site	Samples analyzed	Lab no.	IS*6110* marker	*katG* ^463^	*gyrA* ^95^	*katG* ^203^	*gyrB*(1410)	1977	3352929	2460626	232574
Batta Tcharana	4 vertebrae	YAKa66_A	negative	-	C	-	-	-	-	-	G
		YAKa66_B	positive	C	C	G	-	-	-	-	G
		YAKa66_C	positive	C	C	G	C	G	C	-	G
		YAKa66_D	positive	C	C	-	-	-	-	-	G
Atakh	3 vertebrae	YAKa88_A	positive	C	C	G	C	-	-	-	-
		YAKa88_B	positive	-	C	G	C	-	-	-	-
		YAKa88_C	positive	-	C	G	C	-	-	-	-
Odjuluun 2	1 vertebrae	ODJ_2	positive	C	C	G	C	G	C	C	G
Buguyekh 3	1 vertebrae	BUG_3	positive	-	-	-	-	-	-	-	-

Positions and alleles are relative to the plus strand on the *M. tuberculosis* H37Rv genome sequence, GenBank accession no. NC_000962.3, as described in Bouakaze et al. [32].

Replication of SNapShot results was evaluated by sequencing the SNP positions (*katG* ^203^, *katG* ^463^, *gyrA* ^95^ and *gyrB*(1410) for the three subjects with positive SNP bases (Table 4). On average at least two or more sequences were obtained to validate the SNapShot results, demonstrating the presence of determinant SNPs at target positions (Table S3). *GyrB* sequences of good quality could not be produced for one subject (Batta Tcharana), but the amplified segments for the other three subjects were analyzed and repeatedly confirmed the target SNP positions. Mutations in other positions on the same segment (for example on *gyrA95* segment for three samples; Table S3) were also confirmed on both strands and from different extracts when available. However, information on deviating sequences focuses on some positions in a very short region, and no specific treatment of these sequences is available except the confirmation that the muted positions are on both strands (Table S3).

Only samples with bone TB lesions were tested for MTBC DNA genotyping to identify the type of strains which had infected these Siberian subjects. Samples without typical lesions have not yet been tested for MTBC markers.

## Discussion

In this study we confirmed several cases of bone tuberculosis by SNP genotyping to show that ancient samples had successfully amplified the IS*6110* region. The sample consisted of 140 frozen bodies, from Yakutia (eastern Siberia), dating from the 16^th^ –19^th^ centuries, in an exceptional state of preservation due to their burial in the permafrost. For a long time this region had been isolated from contact with European populations, and it was not until the beginning of the 17^th^ century that first contacts were made with Europeans, including Slavic Russians. The ancient strain of tuberculosis was identified to understand the impact of tuberculosis on the immunologically naïve Yakut population.

### The evolution of tuberculosis frequencies through times

In this study, the two oldest archaeological subjects with bone tuberculosis date from the late 17^th^ century (after 1689), and originate from Central Yakutia where the Europeans settled first [34]. In our sample no cases of tuberculosis were found in subjects from the 19^th^ century (Table 2). If the chronological development of the sample over time and the distribution of bone tuberculosis cases are correlated, we find a typical rise, peak and decline in the number of tuberculosis cases over about one hundred years (Figure 4). This distribution mirrors the theoretical plot of high mortality from tuberculosis over time when tuberculosis first emerges in a human population group [35]. From a theoretical point of view [36], the shape of the epidemic curve for tuberculosis is the same as that for any other infectious disease, but with much longer time span [37]. The rate of bone tuberculosis differs among populations, but it represents less than 25% of the maximal rate of extra pulmonary tuberculosis [38], [39]. The distribution of cases with bone lesions compatible with tuberculosis is, however, very specific, and it could reflect the evolution of tuberculosis frequencies in the population.

### Historical and epidemiological data

Yakuts knew the signs of pulmonary tuberculosis prior to the 20^th^ century, and were able to differentiate it from other respiratory diseases [40], [41]. During the ‘golden age’ of Yakutia, between 1689 and 1728 [4], the prevalence of subjects in our sample with bone tuberculosis at death increased from 11.1% to 14% (Figure 4). We can therefore suggest that at the time death rates from TB (both pulmonary TB and extra-pulmonary TB) were superior to this percentage. The spread of TB could have been linked to the confinement of subjects in their homes during winter [42], [43]. As diagnosed by bone lesions (Figure 3) and confirmed by molecular testing (Table 4), and since the strain identified in our samples may be of European origin (see below), it is highly likely that the disease appeared just a few years after the first contact with Europeans in the early 17^th^ century. Periods of colonization have been favorable circumstances for the spread of pathogenic agents and the emergence of infectious diseases, for example during the conquest of the New World in the early 17^th^ century [44]–[46] and with the settlement of Japanese islands by migrants from the Asian continent 2000 years ago [47]. Likewise, the history of Siberia is marked by the impact of infectious diseases during the Russian penetration and by the exploration of Siberia [34], [48]. The study of European subjects buried in the pioneer town of Krasnoyarsk in Southwest Yakutia, showed that bone tuberculosis also existed in this population during the same period, but at a lower frequency [49]. We did not find cases of bone tuberculosis in samples dating from the 19^th^ century; it may have become a rare disease at this time or changed its clinical form [50]. During the 19^th^ century, a relatively low occurrence of tuberculosis in the Yakut countryside was reported by the medical profession [12], [40]. The re-emergence of tuberculosis in the late 19^th^ and early 20^th^ centuries appears to be linked to the arrival of new migrants, including political exiles and prisoners, killing over 24–63% of subjects between 1900 and 1904 [11]. There were also many incidences of tuberculosis in the 1930s [10], [51], when the creation and collectivization of villages probably favored the spread of the disease.

### Molecular identification of the tuberculosis strain

As discussed above, the Eastern Siberian region had been isolated for a long period, during which time autochthonous populations, including Yakuts, did not have contact with Europeans. First commercial exchanges began from the 17^th^ century, following contact with Slavic Russians. It is worth asking whether the TB strain affecting the native population existed in Yakutia prior to this time and emerged due to the deterioration of Yakut standard of living. Alternatively, it could have been an exogenous TB strain transmitted by the new settlers that infected an immunologically naïve Yakut population.

*M. tuberculosis* species strains are classified into genotypic lineages and families using different genetic markers, and their global distribution can be studied at the country, sub-regional and continental levels [52]–[55]. These genetic markers allow to study the evolution, speciation, descent and migration of the bacterium and its host [52], [55]–[62]. Among the large-scale groupings, those most recognized are: (i) an established rough classification of Sreevatsan et al. [31] that subdivides *M. tuberculosis* into three Principal Genetic Groups (PGG) based on SNPs in *gyrA95* and *katG463*; and (ii) a more recent classification, based on large unique genomic deletions that subdivides *M. tuberculosis* sensu stricto into six lineages [54]. Both classifications correlate with each other and with *M. tuberculosis* families defined by other markers, such as spoligotypes and VNTR, although at different level of resolution [1].

The *M. tuberculosis* strains currently circulating in the Russian Federation mainly belong to the: (i) East Asian lineage, principally known for the Beijing genotype, which falls within the otherwise large, heterogeneous and more evolutionarily ancient PGG-1; and (ii) Euro-American lineage that belongs to PGG-2 and includes several families, such as LAM (Latin-American Mediterranean), Haarlem, Ural, X, T, and S [52]. The Beijing family is considered omnipresent and epidemic in Russia. It accounts for 30 to 60% of local *M. tuberculosis* populations [57], [63], [64]. Moreover, across Siberia and Russian Far East, data are available [63]–[66], and show regional variation in the prevalence of the Beijing genotype, even in neighboring areas (e.g., 56% in Irkutsk versus 31% in Yakutia) [63].

Here, we demonstrated that the ancient *M. tuberculosis* DNA isolate recovered in Yakut mummies of the 17^th^ and 18^th^ centuries (i) is not related to the *M. bovis* lineage and (ii) belongs to the genetic group PGG-2. This latter finding is remarkable in view of the absolute or relative predominance of the Beijing family (i.e., PGG-1) across present day Russian Federation. The Beijing family probably originated in northern China at least 2000 years ago [58], or even earlier [62]. There are different hypotheses regarding when it first entered present-day Russia and Siberia: the medieval entry hypothesis of Mokrousov [58] versus the much more recent 20^th^ century scenario of Sinkov [60]. The former hypothesis is not supported by our ancient DNA results: we did not find the *M. tuberculosis* Beijing genotype in the 17–18^th^ century samples from Yakutia. We cannot, however, completely rule out its presence in Yakutia during the 17^th^ century based on the analysis of only one ancient DNA sample. In other words, the absence of the Beijing genotype in our ancient DNA sample may be due to its true absence in that ancient setting or simply reflects its permanently low prevalence in Yakutia, at present time [63] and three centuries ago.

The analysis of the additional eight SNPs permitted us to classify the DNA isolate into the SNP cluster group SCG-5, that includes LAM, S, and some of the T and H family genotypes [53], and falls within the large and heterogeneous Euro-American lineage. Indeed non-Beijing genetic families in the present-day *M. tuberculosis* population in Yakutia are LAM, Ural, S, T, and some of them appear to be prevalent only in this area, such as a clonal group within the S-family [63]. Interestingly, some of these families fall within the SNP cluster group SCG-5. Thus, the 18^th^ century *M. tuberculosis* DNA in Yakutia may represent a strain of European origin. However, complete profiles of the three other subjects are absent, and other strains could circulate in Yakutia at the same time, as demonstrated in the recent study of 200-years old Hungarian mummy [67].

### Emergence of TB in naïve populations

The impact of tuberculosis on autochthonous populations may have been underestimated [43], or labeled as pulmonary tuberculosis [13], [40]. Our study demonstrates that the outbreak of TB in a naïve population, just after contact with Europeans, could have been more devastating than recorded previously. In the case of the Yakuts, the low effective population size [4], [5] could have been at the origin of genetic drift with a significant effect on negative selection; very few resistance genes may have been present at the beginning of the infection. The peak of the TB epidemic could have, however, selected a subset of relatively resistant survivors who were subjected to the chronic pulmonary disease, the most infectious form of the disease. With the elimination of susceptible individuals, an increasing proportion of the population showed resistance to the infection, and it gradually became an endemic chronic pulmonary disease in the 19th century.

### Conclusion

In conclusion, this multidisciplinary study, involving paleopathology, paleomicrobiology, palaeoepidemiology and history, is the first to be conducted with archaeological samples from Siberia. It demonstrates the presence of tuberculosis in an autochthonous population just a few years after the population's first contact with Europeans, and the highest prevalence of the disease to have been observed. The ancient TB strain identified might be of European origin, but its characterization needs to be specified to fully understand its implication in human Yakut population and its phylogeny related to modern TB strains. We can hypothesize that this strain might have exerted some selective pressure on a small population that was subsequently hit by epidemics caused by other MTB strains centuries later. This favored the evolution of TB, which we will continue to study. The sample material provides an excellent opportunity to research the ancestral pattern of the human populations and the presence of pathogens of parasitic and infectious diseases during Siberian historical period from the beginning of its colonization.

## Supporting Information

Table S1 **PCR primers used in this study.**
(DOC)

Table S2 **Evolution of tuberculosis crude prevalence rate (CPR) in Yakut population from the modern to contemporary era.** Prevalence was calculated as the number of individuals presenting the disease divided by the number of individuals in the study population. Crude Prevalence Rate (CPR) was calculated using the whole study population as the denominator. CPR was calculated for each time period, dividing the number of subjects with the disease by the number of individuals in each period. C: Century.
(DOC)

Table S3 **Final MTBC DNA sequences obtained and compared to H37Rv (Sequence view is plus strand).** The characteristic positions for SNPs are in bold.
(DOC)
