# Supplementary material for: Tuberculosis Epidemiology and Selection in an Autochthonous Siberian Population from the 16th-19th Century
Source: PLoS One. 2014 Feb 26;9(2):e89877. doi: 10.1371/journal.pone.0089877 (PMC3935942; doi:10.1371/journal.pone.0089877)
Supplement: Table S1 — PCR primers used in this study. (DOC) [file pone.0089877.s001.doc]

**Table S1: PCR primers used in this study**

| PCR names | PCR primer sequences (5’→ 3’) |  | Amplicon size (bp) | markers | orientation^a^ |
| --- | --- | --- | --- | --- | --- |
| IS-1 | CTCGTCCAGCGCCGCTTCGG  CCTGCAGCGTAGGCGTCGG | IS1  IS2 | 123 | IS*6110* | F |
| IS-3 | TTCGGACCACCAGCACCTAA  TCGGTGACAAAGGCCACGTA | IS3  IS4 | 92 | IS*6110*  Nested PCR | F |
| mixPCR1 | CGGTCGAAACTAGCTGTGAGACAGT  AAGCCGAGATTGCCAGCCTTA | Forward  Reverse | 72 | *katG*^463^ | F |
|  | GTTCACGTAGATCAGCCCCATCTG  ATGAGGTCTATTGGGGCAAGGAAG | Forward  Reverse | 113 | *katG*^203^ | F |
|  | CGAGACCATGGGCAACTACCA  ATTGCCTGGCGAGCCGAAGT | Forward  Reverse | 130 | *gyrA*^95^ | F |
|  | TCAAATCGTTTGTGCAGAAGGTCTGT  CACCAACTCTCGTGCCTTACGTG | Forward  Reverse | 143 | *gyrB*(1410) | F |
| mixPCR2 | TGAGAAGCTCTACGGTTGTTGTTCG  TTTCACCTCACGATGAGTTCGATCC | Forward  Reverse | 131 | 1977 | F |
|  | TCGACGTCCGGGTAGCATTC  GCGTCGCAAGCATCTGACATT | Forward  Reverse | 108 | 3352929 | F |
|  | GGGCTCGCAGCCAGACTTC  ATGATCACGGCGACCCAGAC | Forward  Reverse | 120 | 2460626 | F |
|  | CCACGGCGGGGACAAGAT  AGAAAGGCGCCGCTGTAGG | Forward  Reverse | 81 | 232574 | F |
|  | Neutral sequence (5’→ 3’) | Target specific sequence (5’→ 3’) | Size (nt) | markers | orientation^a^ |
| SBE 1 | CGTGAAAGTCTGACAA | GCCTTAAGAGCCAGATCC | 34 | *katG*^463^ | R |
|  | CCACGTCGTGAAAGTCTGACAA | ACGCGTCGATCTACGACA | 40 | *gyrA*^95^ | F |
|  | ACTAGGTGCCACGTCGTGAAAGTCTGACAA | GCTCATCGCCGAGCCA | 46 | *katG*^203^ | F |
|  | AACTGACTAAACTAGGTGCCACGTCGTGAAAGTCTGACAA | GTTTGTGCAGAAGGTCTGTAA | 76 | *gyrB*(1410) | F |
| SBE 2 | GTGAAAGTCTGACAA | GACTGCCAACGACGAA | 31 | 1977 | R |
|  | TCGTGAAAGTCTGACAA | CTGACATTGGTGCACAAAAC | 37 | 3352929 | R |
|  | ACTGACTAAACTAGGTGCCACGTCGTGAAAGTCTGACAA | GGACCGAGGGTCTGGC | 55 | 2460626 | F |
|  | AACTGACTAAACTAGGTGCCACGTCGTGAAAGTCTGACAA | ACAGGGCAATCACCTCG | 61 | 232574 | F |

^a^ Orientation of primer according to genomic sequence of *M.tuberculosis* H37Rv (NC_000962.3). R, reverse ; F, Forward.

IS*6110* primers are described in [29]. All other primers and all conditions of PCR are described in [32].
