# Supplementary material for: Tuberculosis Epidemiology and Selection in an Autochthonous Siberian Population from the 16th-19th Century
Source: PLoS One. 2014 Feb 26;9(2):e89877. doi: 10.1371/journal.pone.0089877 (PMC3935942; doi:10.1371/journal.pone.0089877)
Supplement: Table S2 — Evolution of tuberculosis crude prevalence rate (CPR) in Yakut population from the modern to contemporary era. Prevalence was calculated as the number of individuals presenting the disease divided by the number of individuals in the study population. Crude Prevalence Rate (CPR) was calculated using the whole study population as the denominator. CPR was calculated for each time period, dividing the number of subjects with the disease by the number of individuals in each period. C: Century. (DOC) [file pone.0089877.s002.doc]

**Table S2:** Evolution of tuberculosis crude prevalence rate (CPR) in Yakut population from the modern to contemporary era.

|  |  | Cases | |  | CPR | |
| --- | --- | --- | --- | --- | --- | --- |
| Period | Duration | Number | 95% CI | Sample size | Value | 95% CI |
| 16-17^th^ C | 200 y | 2 | 0.24-7.22 | 18 | 11.1 | 1.3-40.1 |
| 18th C (1^st^ half) | 50 y | 7 | 2.81-14.42 | 50 | 14 | 5.6-28.8 |
| 18th C (2nd half) | 50 y | 4 | 1.09-10.24 | 34 | 11.7 | 3.2-30.1 |
| 19th C | 100 y | 0 | 0-3.67 | 37 | 0 | 0-9.9 |

Prevalence was calculated as the number of individuals presenting the disease divided by the number of individuals in the study population. Crude Prevalence Rate (CPR) was calculated using the whole study population as the denominator. CPR was calculated for each time period, dividing the number of subjects with the disease by the number of individuals in each period. C: Century.
