# Supplementary material for: Tuberculosis Epidemiology and Selection in an Autochthonous Siberian Population from the 16th-19th Century
Source: PLoS One. 2014 Feb 26;9(2):e89877. doi: 10.1371/journal.pone.0089877 (PMC3935942; doi:10.1371/journal.pone.0089877)
Supplement: Table S3 — Final MTBC DNA sequences obtained and compared to H37Rv (Sequence view is plus strand). The characteristic positions for SNPs are in bold. (DOC) [file pone.0089877.s003.doc]

**Table S3**: Final MTBC DNA sequences obtained and compared to H37Rv (Sequence view is plus strand). The characteristic positions for SNPs are in bold.

*KatG* gene positions: 2153889-2156111; ***KatG^463^*** segment positions: 2154711-2154736.

2154711 2154524 2154736

....|....|....|....|....|.

**NC_000962.3**  CAATCCCGATGCC**C**GGATCTGGCTCT

**Sequence Atakh** **.............C.-----------**

**Sequence Batta Tcharana** **----.........C............**

**Sequene Odjulunn** **----.........C...---------**

*GyrA* gene positions: 7302-9818); ***gyrA^95^*** segment positions: 7556-7644.

7556 7585 7644

|....|....|....|....|....|....|....|....|....|....|....|....|....|....|....|....|....|...

**NC-000962.3** CCCGCACGGCGACGCGTCGATCTACGACA**G**CCTGGTGCGCATGGCCCAGCCCTGGTCGCTGCGCTACCCGCTGGTGGACGGCCAGGGCA

**Sequence Odjulunn ---------------------........C.........G...........G.............------------------------**

**Sequence Batta Tcharana ---------------------........C.........G...........G.....A...............................**

**Sequence Atakh ---------------------........C......A..G...........G.....A.....G.........----------------**

*KatG* gene positions: 2153889-2156111; ***KatG^203^*** segment positions: 2155443-2155507.

2155503

....|....|....|....|....|....|....|....|....|....|....|....|....|

**NC_000962.3**  CACCGCGGCCAGCGGGTTCTCCAGATCCCGCTTACCGCTGTAACGCTCATCGCCGAGCCA**G**GTGG

**Sequence Atakh**  **--------------------------------------------------..........G...T**

**Sequence Batta Tcharana** **............................................................G...-**

**Sequence Odjulunn** **----------------------------------------------..............G....**

*GyrB* gene positions: 5240-7267; ***GyrB*(1410)** segment positions : 6404-6440

6406

....|....|....|....|....|....|....|..

**NC_000962.3** AA**C**GAACAGCTGACCCACTGGTTTGAAGCCAACCCCA

**Sequence Odjulunn**  **..C........................----------**

**Sequence Atakh**  **..C........C..G........C..-----------**
